# Systematic characterization of lncRNAs' cell-to-cell expression heterogeneity in glioblastoma cells

**DOI:** 10.18632/oncotarget.7580

**Published:** 2016-02-23

**Authors:** Dekang Lv, Xiang Wang, Jun Dong, Yan Zhuang, Shuyu Huang, Binbin Ma, Puxiang Chen, Xiaodong Li, Bo Zhang, Zhiguang Li, Bilian Jin

**Affiliations:** ^1^ Institute of Cancer Stem Cell, Cancer Center, Dalian Medical University, Dalian, 116044, Liaoning, P.R. China; ^2^ Department of Neurosurgery, The Second Hospital of Dalian Medical University, Dalian, 116023, Liaoning, P.R. China; ^3^ Department of Obstetrics and Gynecology, The Second Xiangya Hospital of Central South University, Changsha, 410011, Hunan, P.R. China

**Keywords:** GBM, lncRNA, heterogeneity, single-cell RNA-seq, self-organizing map

## Abstract

Glioblastoma (GBM) is the most common malignant adult brain tumor generally associated with high level of cellular heterogeneity and a dismal prognosis. Long noncoding RNAs (lncRNAs) are emerging as novel mediators of tumorigenesis. Recently developed single-cell RNA-seq provides an unprecedented way for analysis of the cell-to-cell variability in lncRNA expression profiles. Here we comprehensively examined the expression patterns of 2,003 lncRNAs in 380 cells from five primary GBMs and two glioblastoma stem-like cell (GSC) lines. Employing the self-organizing maps, we displayed the landscape of the lncRNA expression dynamics for individual cells. Further analyses revealed heterogeneous nature of lncRNA in abundance and splicing patterns. Moreover, lncRNA expression variation is also ubiquitously present in the established GSC lines composed of seemingly identical cells. Through comparative analysis of GSC and corresponding differentiated cell cultures, we defined a stemness signature by the set of 31 differentially expressed lncRNAs, which can disclose stemness gradients in five tumors. Additionally, based on known classifier lncRNAs for molecular subtypes, each tumor was found to comprise individual cells representing four subtypes. Our systematic characterization of lncRNA expression heterogeneity lays the foundation for future efforts to further understand the function of lncRNA, develop valuable biomarkers, and enhance knowledge of GBM biology.

## INTRODUCTION

Glioblastoma (GBM), the most common and aggressive form of primary malignant brain tumor in adults, is one of the most lethal human cancers [1]. Despite the advance of clinical standard treatment usually comprising surgery, radiation and chemotherapy over past decades, the median survival for patients with glioblastoma has remained less than two years [2]. It is believed that the dismal prognosis is, at least partially, attributed to tumor heterogeneity that was first demonstrated by histopathological discoveries [3]. GBM is found to be highly cytologically pleomorphic. Its constituent cells not only possess a high degree of variation in size and shape but also usually have large bizarre nuclei or are multinucleated [4].

GBM has now manifested its heterogeneous nature in many ways. It is, however, becoming increasingly clear that intratumoral genetic heterogeneity is central to GBM biology, potentially posing a great challenge to effective treatment [5]. Originally, intratumoral heterogeneity has been verified via the analysis of bulk tumors revealing regional copy number variation (in *EGFR*, *PDGFRA* and *PTEN*), heterogeneous somatic mutations (in *TP53*) or gene expression difference (in *MGMT*) [6, 7]. Further supportive evidence comes from the observation that spatially distinct fragments sampled from the same tumor corresponded to different GBM molecular subtypes [7]. These findings represent an important step toward understanding intratumoral heterogeneity, but they deserve closer scrutiny at higher resolution because each cell within a single tumor possibly possesses a unique gene expression signature under specific conditions.

Single-cell analysis allows an accurate recapitulation of cell-to-cell variations instead of the stochastic average masked by bulk measurements [8, 9]. Recently developed single-cell RNA-sequencing (RNA-seq) has enabled highly parallel transcriptome-wide analysis of hundreds of thousands of cells, providing the high-resolution landscape of the heterogeneity of single cells within a population [10]. Using the method, a systematic profiling of a large number of individual cells from five primary GBM tumors revealed deep insights into cell-to-cell variability in expression of diverse transcriptional programs [11].

The above-mentioned studies mainly focused on the analysis of protein-coding transcripts, probably because most of their translated proteins are important signaling molecules. Indeed, a new class of transcripts, long noncoding RNAs (lncRNAs) can exert their effects through mechanisms such as chromatin remodeling, *cis* regulation at enhancers and post-transcriptional regulation of mRNA processing [12]. Thus, they have been proposed as key mediators of diverse biological processes including cell pluripotency and tumorigenesis [12-14]. Currently, accumulated evidence demonstrates that some lncRNAs, often aberrantly expressed in GBM, have been implicated in histological/molecular subtypes and malignant phenotypes, thereby possessing potentials as biomarkers for diagnosis and prognosis, and as therapeutic targets [15-21].

Obviously, the cell-to-cell variability of lncRNAs merits deeply exploration to further uncover the transcriptional heterogeneity in cancer. Here we used a large set of publicly available single-cell transcriptome data from five primary GBMs and two glioblastoma stem-like cell (GSC) lines to comprehensively interrogate the expression profiles of 2,003 lncRNAs in 380 cells. By utilizing the self-organizing maps (SOMs), we extracted and visualized the lncRNA expression dynamics of individual cells from each tumor and from each cell line. Based on lncRNAs generating multiple splice variants and those involved with stemness and molecular subtypes, detailed analysis of their expression patterns epitomized the fundamental properties of lncRNAs' cell-to-cell expression heterogeneity, providing a new starting point for further understanding the role of lncRNAs in gliomagenesis, developing valuable biomarkers and identifying novel treatment targets.

## RESULTS

### Identification of lncRNAs in single cells from GBM tumors and GSC lines

**Figure 1:**
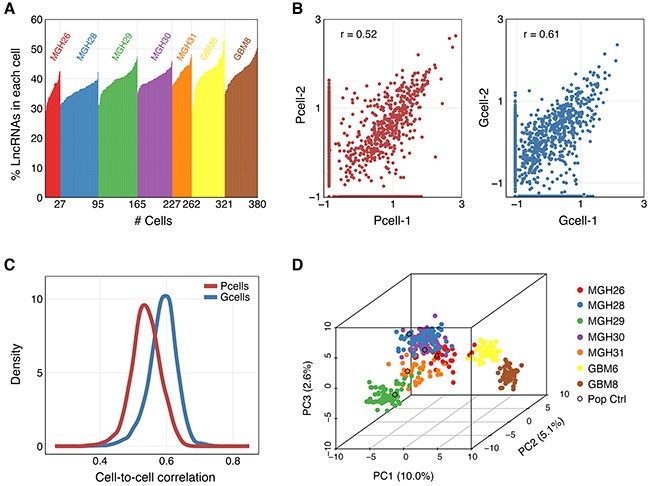
Characterization and correlation between single cell profiles of selected lncRNAs **A.** Percentages of 2,003 selected lncRNAs expressed in each of the single cells from five tumors and two GSC lines. **B.** Scatter plot of normalized lncRNA gene expression values for two randomly selected cells in MGH31 (Pcell, left) and GBM8 (Gcell, right). **C.** Distribution of correlation coefficients for all single cell pairs from the same primary tumor (Pcell, r~0.40-0.65) or GSC line (Gcell, r~0.45-0.75). **D.** Principal component analysis (PCA) of 380 single-cell lncRNA transcriptomes using 500 lncRNAs with the greatest variance among the libraries.

We reanalyzed a previously reported transcriptome dataset that profiled 576 single cells isolated from five primary GBMs (MGH26, 28, 29, 30, 31), 96 resequenced MGH30 cells (MGH30L), 192 single cells from two GSC lines (GBM6 and GBM8) and 11 population samples (five controls for each tumor, three GSC cultures and their corresponding differentiated tumor cell cultures) [11]. We discarded poor-quality cells and transcripts with low coverage, focusing on 2,003 lncRNAs quantified in 262 cells from five tumors, 118 cells from two GSC lines and population samples (Supplementary Table S1). Percentages of these lncRNAs expressed in each of the single cells from five tumors and two GSC lines were shown in Figure 1A. Frequency distribution of individual lncRNAs in each tumor was indicated in Supplementary Figure S1. Individual cells showed the highest correlation with each other within the same tumor or GSC line (Supplementary Figure S2). The two GSC lines were also highly correlated to each other. Additionally, the correlation coefficients between individual cells from the same primary tumor or GSC line were within a wide range (Figure 1B, 1C), suggestive of intratumoral heterogeneity. To analyze lncRNA transcriptional interrelationships among the selected cells, we performed principal component analysis (PCA). The PCA revealed that despite most cells clustered by tumor of origin, some of the cells from one tumor interspersed among the transcriptional space of other tumors (Figure 1D). Moreover, the transcriptional diversity within each tumor was clearly higher than that observed in the two established GSC lines (Figure 1D).

### Overall characterization of lncRNA expression patterns

**Figure 2:**
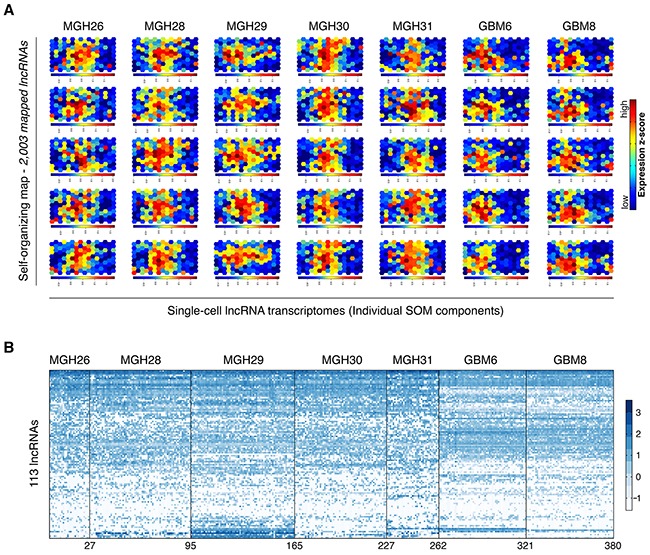
Overview of lncRNA expression dynamics at single-cell level **A.** The self-organizing map (SOM) was used for analysis of lncRNA transcriptome. A SOM component represents one visualized single-cell lncRNA transcriptome. Five representative SOM components are shown for each tumor sample and each GSC line. **B.** Hierarchical clustering of 113 lncRNAs across five tumor samples and two GSC lines. These lncRNAs were derived from a union of five sets of the 50 most abundant lncRNAs in individual population controls.

To obtain an overview of lncRNA expression dynamics, we compiled lncRNA expression data of the tumor samples and GSC lines, and normalized them for constructing the SOM that is capable of exhibiting similarity relationships in a two-dimensional heat map in which spatial neighborhood reflects expression pattern similarity [22]. We mapped 2,003 lncRNAs onto a SOM to evaluate lncRNAs' cell-to-cell variation. LncRNAs with most similar expression patterns were clustered as one set, which was symbolized by a hexagonal unit of the SOM. Individual units were located in the same fixed positions across all single-cell components. A component of the SOM represented one visualized single-cell lncRNA transcriptome (Figure 2A).

We next examined single-cell lncRNA expression profiles using a hierarchical clustering analysis based on 113 lncRNAs from a union of five sets of the 50 most abundant lncRNAs in individual population controls. As shown in Figure 2B, there existed extensive cell-to-cell variability at the lncRNA transcriptional level regardless of primary tumors or GSC lines.

### Coordinate expression of lncRNAs and protein-coding genes

**Figure 3:**
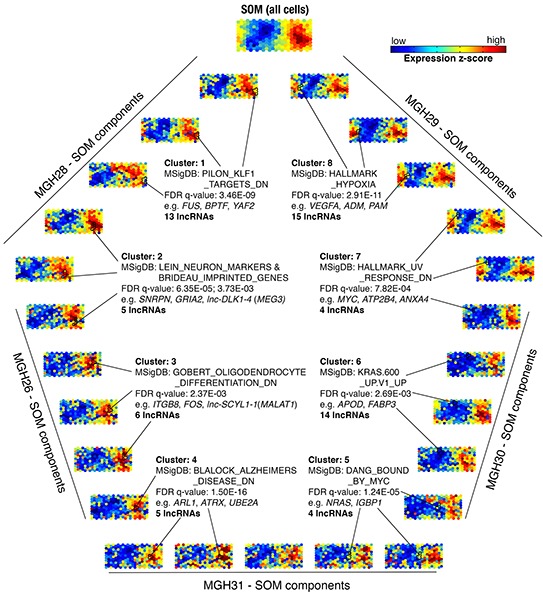
Analysis of coordinately expressed lncRNAs and protein-coding genes The self-organizing map (SOM) was used for analysis of transcriptome composed of 2,003 lncRNAs and 5,145 protein-coding genes. Single-cell transcriptomes were depicted as individual components of the SOM. Five representative SOM components are shown for each tumor sample. A large-scale SOM (top) was constructed based on average of expression z-scores across all 262 cells from five primary tumors. Eight clusters are outlined in black. Significantly enriched Molecular Signatures Database (MSigDB) gene sets for indicated clusters are shown (FDR q-value).

Because the SOM can define sets of coordinately expressed genes and has been used for inferring possible functions of lncRNAs clustered with annotated protein-coding genes [22], we determined 5,145 protein-coding genes and mapped them with 2,003 lncRNAs onto a new SOM (Figure 3). The Molecular Signatures Database (MSigDB) [23] was used to determine the enriched annotations for clustered genes (Supplementary Table S2). Typically, Cluster 8 composed of three sets contained *VEGFA* and numerous hypoxia-related genes, including *PAM*, *ADM*, and *ATF3*, which was significantly enriched for the MSigDB's gene set “HALLMARK_HYPOXIA” (FDR q-value: 2.91×10^−11^). Fifteen lncRNAs were coordinately expressed with these genes, suggesting their possible involvement in the hypoxia signaling pathways. In Cluster 2, five lncRNAs were grouped with 16 genes that were significantly enriched for the gene sets “LEIN_NEURON_MARKERS” and “BRIDEAU_IMPRINTED_GENES” (FDR q-value: 6.35×10^−5^ and 3.73×10^−3^, respectively). Of these, *lnc-DLK1-4* (often called *MEG3*, a well-characterized tumor suppressor) could be annotated to these two gene sets, supporting the utility of this SOM analysis on initial prediction for potential roles of unknown lncRNAs.

### Cell-to-cell variation in splicing patterns of lncRNAs

**Figure 4:**
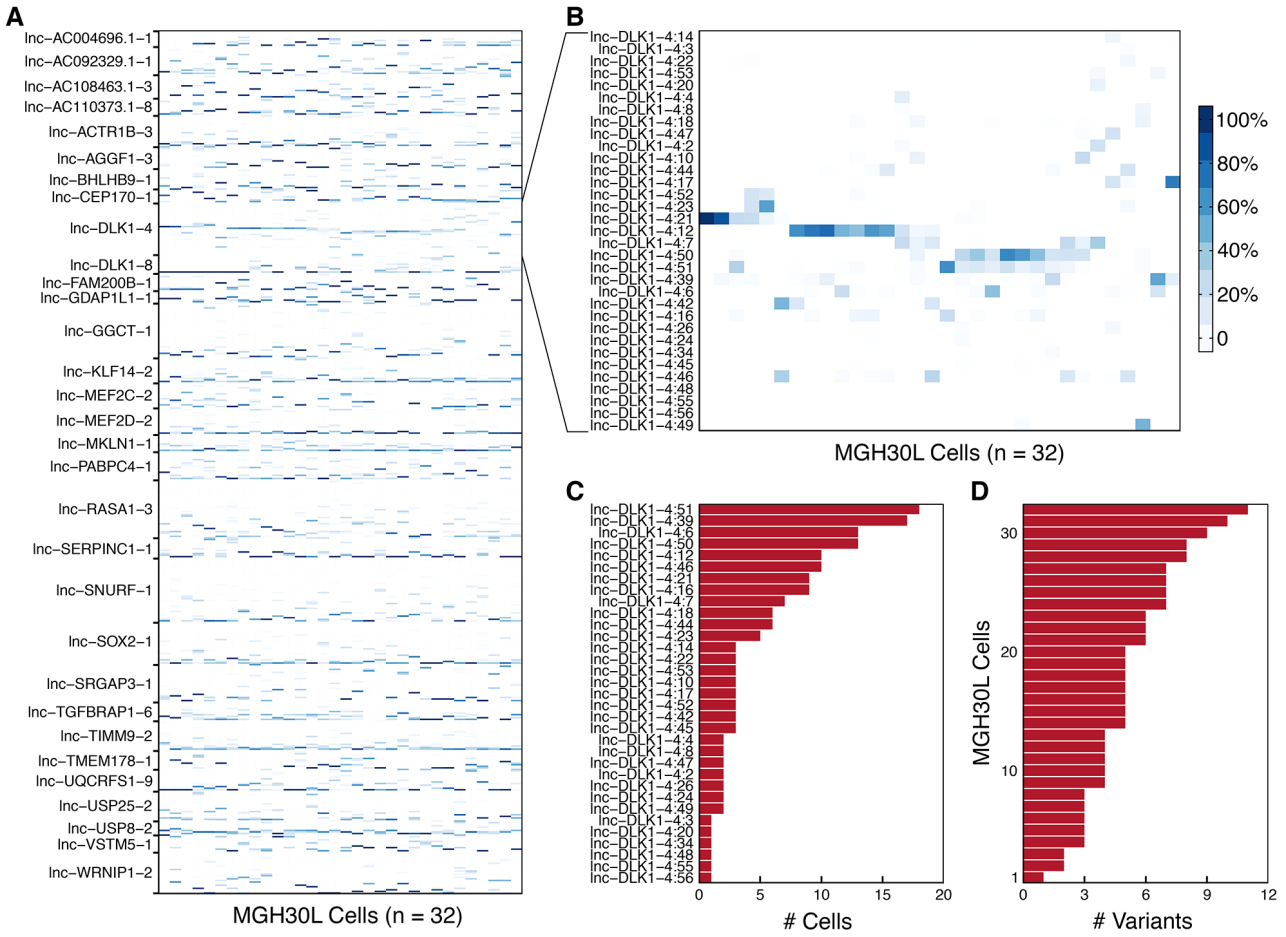
Variation in splicing patterns of lncRNAs in single cells **A.** Heatmap depicts the within-gene relative abundances of 552 splice variants corresponding to the selected 31 lncRNA genes among the 32 individual cells from MGH30 tumor sample. **B.** Heatmap displays the obvious heterogeneity in the splicing patterns of *lnc-DLK1-4* (*MEG3*) across the 32 individual cells within MGH30. **C.** The frequency distribution of 33 *lnc-DLK1-4* variants in the single cells from MGH30 is indicated. **D.** The number of individual *lnc-DLK1-4* splice variants expressed by each cell from MGH30 is shown.

To examine whether single-cell heterogeneity is also present in splicing events of lncRNAs, we mapped 32 MGH30L resequenced data with long reads to the reference transcriptome to call splice variants and estimate their relative abundances. Subsequently, 31 lncRNA genes having at least 10 variants were selected to determine expression patterns of their variants in each cell. After filtering those variants without expression levels, 552 splice events of lncRNAs were detected among individual cells (Figure 4A). We observed that these lncRNAs tended to express multiple variants simultaneously, and alternative variants from the same lncRNA were not always expressed at similar levels across individual cells. We also found that one variant dominated in a small fraction of single cells, and 15 of the 31 lncRNAs had more than two dominant variants (Supplementary Figure S3).

As an exemplar of alternative splicing of lncRNAs, *lnc-DLK1-4* expressed 33 variants and displayed different splicing patterns across the 32 individual cells (Figure 4B, Supplementary Figure S4). There were four dominant variants expressed in the single cells. Obviously, lnc-DLK1-4:21 and lnc-DLK1-4:12 displayed almost mutually exclusive expression patterns. By contrast, lnc-DLK1-4:50 and lnc-DLK1-4:51 were preferentially co-expressed with each other. As shown in Figure 4C, some variants occurred more frequently than others. In particular, 6 variants (lnc-DLK1-4:3, -4:20, -4:34, -4:48, -4:55, and -4:56) were observed only once, whereas variant lnc-DLK1-4:39 was observed 17 times and variant lnc-DLK1-4:51 was observed 18 times. In addition, only one single cell expressed a variant and the remaining 31 individual cells expressed two to 11 variants (Figure 4D). Taken together, these observations suggested that the changes in the frequency and proportion among variants directly reflected the cell-to-cell heterogeneous nature of splicing patterns, which might alter lncRNAs' functions and interactions with their partners, and thus probably contribute to gliomagenesis.

### Analysis of stemness-specific lncRNAs and their heterogeneous expression

GBM is one of the first solid tumors that are experimentally confirmed to possess cancer stem cells (CSCs) [24]. There have been some initial studies that strongly suggested the highly dysregulated expression of lncRNA in GSCs, which might have decisive effects on the formation of certain malignant phenotypes in GBM [14]. Using six population-level sequencing data of GSC and corresponding differentiated glioma cell (DGC) cultures from three tumors (MGH26, 28 and 31), we identified a consensus set of 31 differentially expressed lncRNAs standing for a stemness signature (Supplementary Table S3). These lncRNAs were found to be significantly upregulated in GSCs when compared to DGCs. The most striking upregulation was observed for *lnc-SCYL1-1*, *lnc-C1orf35-2*, and *lnc-NDUFS6-6* (*p*-value < 0.01).

**Figure 5:**
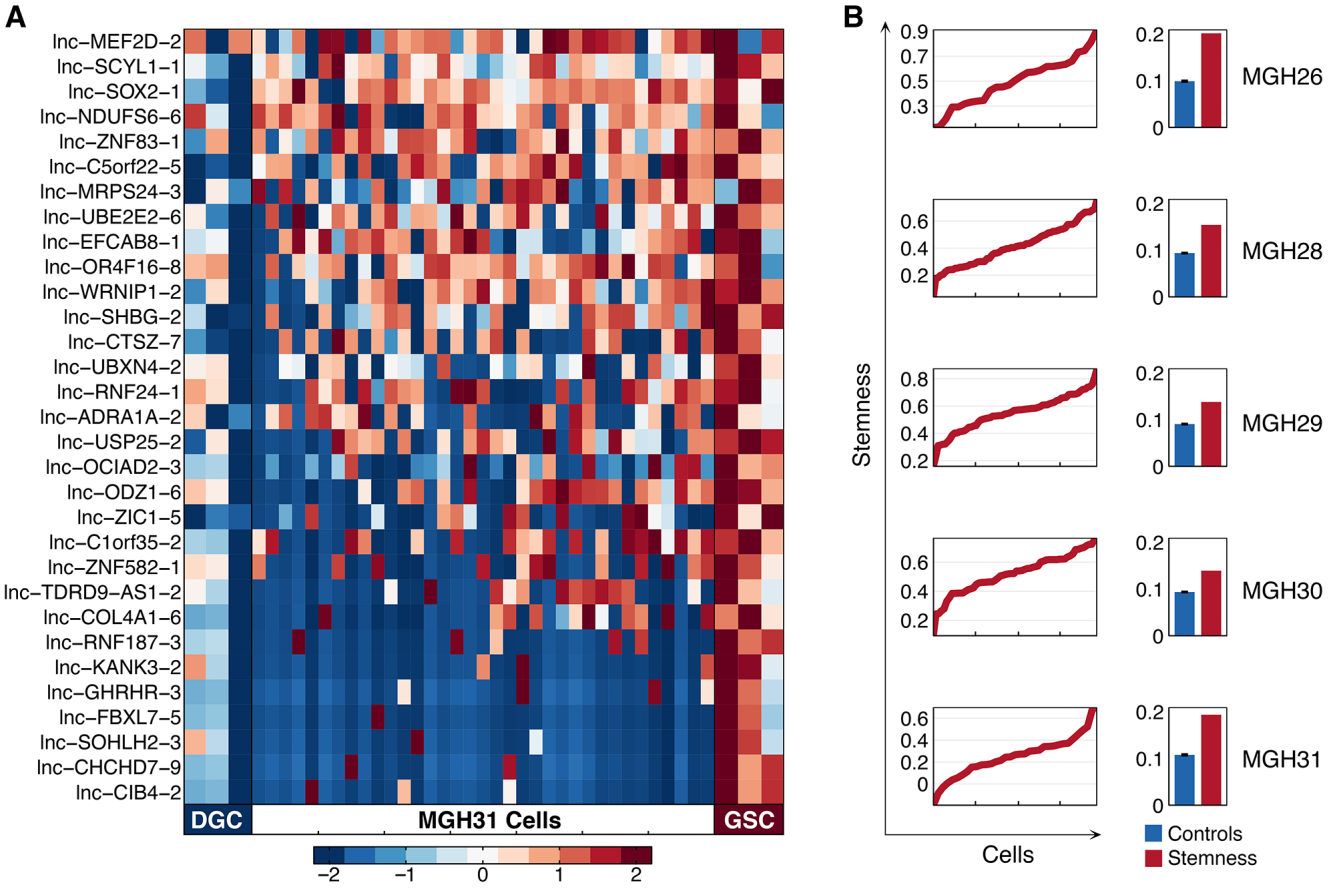
Transcriptional features of stemness-specific lncRNAs in single cells **A.** Heatmap depicts expression of the 31 lncRNAs representing the stemness signature in differentiated glioma cell (DGC) cultures (left columns), glioblastoma stem-like cell (GSC) cultures (right columns) derived from three tumors (MGH26, 28 and 31), and in 35 individual cells from MGH31 (middle). **B.** Plot depicts stemness score (*y* axis) calculated from expression levels of the 31 stemness-specific lncRNAs in individual cells from each tumor (*x* axis) ordered by score. Bar graphs show the overall variance (*y* axis, SD) in the stemness score (red) and the average variance of simulated control lncRNA sets (blue), demonstrating the significance of the stemness-gradient.

We next displayed the expression patterns of stemness signature across single cells within each tumor sample (Figure 5A, Supplementary Figure S5). Notably, the high expression of *lnc-MEF2D-2*, *lnc-SCYL1-1*, *lnc-SOX2-1* and *lnc-NDUFS6-6* was concurrently observed in a large fraction of cells. To further characterize tumor cells with stemlike or differentiated phenotypes, we plotted a graph of the stemness-differentiation gradient to assess cell-state hierarchies of the five individual primary tumors (Figure 5B). Obviously, stemness axis was occupied continuously, reflecting a continuum of intermediate cellular states within a primary tumor, which resulted from the dynamic regulatory program involved with the stemness.

### Heterogeneous expression of known subtype-specific lncRNAs

In GBM, some of the lncRNAs have been identified as being clinically relevant by means of integrative analysis for genomic data sets and clinical information [16]. We obtained one lncRNA list specific to molecular subtypes. After removing those lncRNAs not in our 2,003 lncRNA library, 31 lncRNAs with greatest variance were used to construct heat maps for subtypes (Supplementary Table S4).

**Figure 6:**
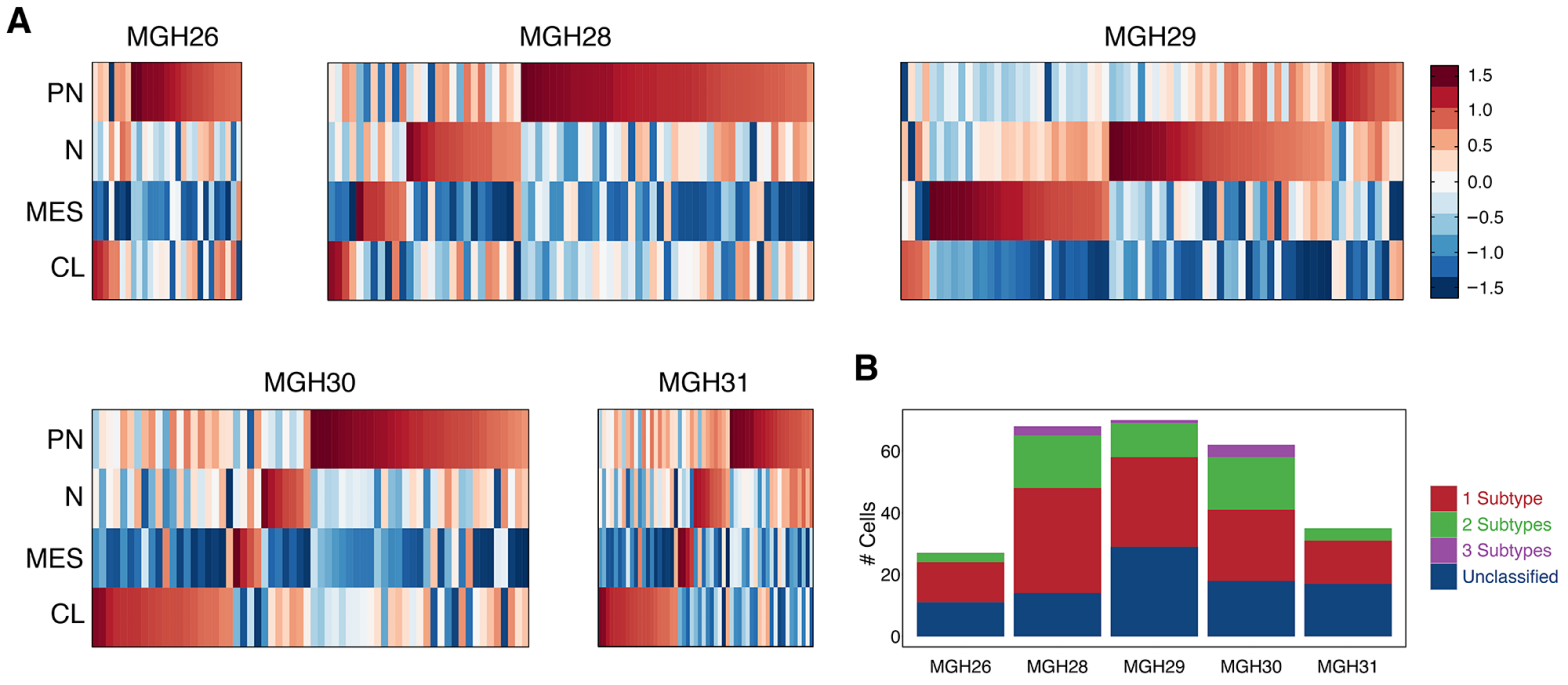
Expression patterns of GBM subtype-specific lncRNAs in individual tumors **A.** Heatmap depicts average expression of classifier lncRNAs for each subtype (rows) across all single cells grouped by tumor (columns). PN: proneural; N: neural; MES: mesenchymal; CL: classical. Each tumor is in a hybrid state, containing some cells representing different subtypes. **B.** Bar graphs depict the number of single cells possessing different quantity of subtypes in each of the five tumors.

The classification scheme established by The Cancer Genome Atlas (TCGA) defines four GBM subtypes: proneural, neural, classical and mesenchymal [25]. Therefore, four corresponding classifier lncRNA sets (Supplementary Table S4) extracted from the subtype-specific lncRNA list were used to examine whether individual cells in a tumor vary in their classification. A previous report has confirmed that the tumors in this study are classified as proneural (MGH26), classical (MGH30), or mesenchymal (MGH28 and MGH29) subtypes [11]. Nevertheless, Figure 6A showed that all five primary tumors were hybrid and comprise individual cells representing different subtypes regardless of the dominant subtype of the tumor. Interestingly, in each tumor, there were some cells that conformed to a proneural subtype. Moreover, more than one subtype can coexist in a fraction of cells within individual tumors (Figure 6B). We also revealed that the classification determined by lncRNAs for all 262 individual cells had the average ~77% consistency with that by protein-coding genes.

## DISCUSSION

GBM, as an archetypal example of a heterogeneous cancer, is well worthy of being considered for full assessment of expression changes in lncRNAs at the single-cell level. This is because compelling evidence shows many lncRNAs play an essential role in gliomagenesis [17, 21] and in addition, there exist copious single-cell RNA-seq transcriptome data [11]. Through deeply mining these public data, we demonstrated that the expression of lncRNAs was very heterogeneous across all GBM single cells. The heterogeneous behavior of lncRNAs was further confirmed to occur at different levels of tumor pathogenesis and manifestations, including RNA splicing patterns, cell stemness, and molecular subtypes, and was reminiscent of the high variability between individual cells in terms of genetic and expression changes in signaling and regulatory pathways [11, 26].

A powerful exploratory tool is the prerequisite to extract the maximum information from global expression analysis and to display the dynamic changes of lncRNA expression across different single cells. The SOM has been proven successfully in the analysis of lncRNAs' cell-to-cell variation during somatic cell reprogramming [22]. It is also noteworthy that many studies have long demonstrated the efficacy of SOM as a useful approach for gene expression clustering [27-31]. Moreover, the SOM outperforms classic two-way hierarchical clustering and even *k*-means under certain conditions (e.g. for larger numbers of clusters) [32]. As an unsupervised learning neural network paradigm, the SOM can project high-dimensional data to a lower dimension representation scheme while preserving essential information [33]. As such, based on expression similarity, lncRNAs could be parsed into individual sets, each of which was visualized as one unit of the SOM (Figure 2A). This allows capture of unique gene expression patterns for all single cells at specific states and thus makes it easy to unveil and observe inherent intratumoral heterogeneity through comparing dynamic transcriptional changes between featured gene sets, namely the most visually prominent units within SOMs.

The majority of the lncRNAs used in our analysis are still not functionally annotated. LncRNAs interact extensively with protein-coding genes through various ways such as direct binding and miRNA-mediated competing endogenous RNA (ceRNA) cross-talk to construct regulatory networks [34, 35], in which the expressions of genes acting in synergy are often highly correlated to each other. A great variety of single-cell transcriptomes from a population in a sense provide the opportunity for more finely examining the correlation between coordinately expressed genes. It has been confirmed that gene clusters defined by the SOM can effectively group coordinately expressed lncRNAs and protein-coding genes in the context of analysis of single-cell RNA-seq data [22]. These clusters contain protein-coding genes that tend to be significantly enriched for known functional categories, which are further utilized for inferring potential functions of unannotated lncRNAs in the same cluster [22]. Therefore, we built a larger SOM to curate 7,148 genes including all 2,003 lncRNAs into different clusters. As a result, some of them were identified to significantly correlate with specific functional roles or critical signaling pathways for GBM. In particular, there were three clusters that enriched genes linked to hypoxia, *KRAS* and *MYC* pathway signatures respectively. Of note, further studies are needed to determine the meaningfulness of lncRNAs to different signatures.

Alternative splicing in cancer leads to the production of antagonistic variants that can contribute to tumor cell survival, growth, invasion, and metastasis [36]. Although lncRNA genes tend to have fewer exons than protein-coding genes [12], considerable splicing events were observed for the selected lncRNAs across single cells within a given tumor (Figure 4A). Moreover, splicing patterns of lncRNAs displayed many basic characteristics, which were consistent with the previous observation for protein-coding genes [37]. Because cancer-specific splice variants are likely to be considered as potentially versatile biomarkers as well as therapeutic targets [38, 39], identification of splicing events for lncRNA is of practical importance. A recent study has shown that a transcript variant of *lnc-IRX3-4* (often called *CRNDE*), most significantly upregulated in gliomas, promotes tumor cell growth and invasion, indicating that it may serve as a novel therapeutic target [40]. Nevertheless, our analysis clearly disclosed that different splicing variants of lncRNAs including *lnc-DLK1-4* were heterogeneously expressed across individual tumor cells (Figure 4). These findings suggest that cell-to-cell variation in splicing patterns should be taken into account when accurately identifying the function of lncRNAs and efficiently screening and selecting reliable biomarkers or therapeutic targets.

The origin of cellular heterogeneity in GBM is believed to occur partly from differentiation of GSCs, a small subpopulation of the cancer cells that represent a reservoir for the drug resistance and tumor recurrence [6, 41]. Our study confirmed that the 31 lncRNAs were significantly upregulated in GSCs relative to DGCs. Some of the lncRNAs have been reported to be stemness-specific in other types of tumors [17, 42]. Notably, *lnc-SCYL1-1*, often called *MALAT1*, is a crucial factor for enhancing stem cell-like phenotypes in pancreatic cancer [43]. Although the role of *lnc-SCYL1-1* in GSCs has yet to be examined, its expression positively correlates with the malignant status and poor prognosis of glioma [17]. In addition, *lnc-SOX2-1*, also called *SOX2-OT*, can positively promote the transcription of *SOX2* gene (one of the major regulators of pluripotency) and is dysregulated in esophageal squamous cell carcinoma, lung squamous cell carcinoma, and breast cancer [42]. These studies have important implication that the newly identified lncRNAs most probably contribute to the stemness of glioblastoma cells.

Currently, the four subtypes defined by TCGA have extensively been applied to investigating cellular composition of GBM, because they have specific differentiation characteristics linking to alternative cells of origin that is critical for establishing effective treatment regimens [25]. The subtype-specific lncRNAs can classify most of the individual cells within a single tumor, which is highly consistent with the observation on the basis of the expression patterns of protein-coding genes. Indeed, lncRNA expression is more tissue and cell type specific than that of protein-coding genes in cancer [44], supportive of a special research reclassifying glioma into three novel subtypes only depending on lncRNA profiles [45]. In our study, some of the single cells were revealed to possess more than one subtype, indicating they were in the hybrid states. It not only at least partly reflects aberrant developmental programs or interconversion between phenotypic states [11], but also provides the crucial information on the diversity of transcriptional subtypes within a tumor. These will be very useful for the selection of lncRNAs as specific biomarkers in early detection and diagnosis, and for the evaluation of prognosis of cancer.

In conclusion, our systematic characterization of lncRNA expression patterns at the single-cell level lays the foundation for future efforts to better understand the function of lncRNA, develop valuable biomarkers, select therapeutic targets, and enhance knowledge of GBM biology.

## MATERIALS AND METHODS

### Single-cell RNA-seq dataset

RNA expression datasets and the corresponding sample information were downloaded from NCBI GEO DataSets websites (http://www.ncbi.nlm.nih.gov/gds/) with accession no. GSE57872.

### Processing of RNA-seq data

The transcriptome used for mapping contains 80,216 high-confidence lncRNA transcripts corresponding to 48,028 lncRNA genes from LNCipedia 3.0 [46] and all protein-coding genes of Ensembl (version 74) [47]. Bowtie (version 1.1.1) [48] was used to map paired-end 25-bp reads with parameters -n 0 -e 99999999 -l 25 -I 1 -X 2000 -a -m 15. Only uniquely mapped reads were retained and utilized for estimating expression levels of all transcripts by RSEM (version 1.2.19, with default parameters) [49]. TPM values as defined by RSEM were added a value of 1 to avoid zeros and then log2-transformed. The most abundant 7,148 genes including 2,003 lncRNAs were selected according to two strategies, either average log_2_ (TPM+1)>2 across all cells or average log_2_ (TPM+1)>4 in at least one tumor. Consequently, 380 single cell transcriptomes expressing at least half of these 7,148 genes were retained. Finally, to examine relative expression levels of the genes across cells, normalization was performed by subtracting the average expression (log_2_ (TPM+1)) of each gene and dividing by its standard deviation. For splicing pattern analysis, relative expression abundances of all lncRNAs' variants in MGH30L were estimated by RSEM using only reads with no more than one mismatch.

### Principal component analysis and self-organizing maps

Principal component analysis (PCA) was performed with R function *prcomp* and visualized with the R package ‘scatterplot3d’. For SOM construction, the 500 lncRNA genes (from 2,003 lncRNAs) or 2,383 genes (from 7,148 genes) with the greatest variance among the samples were first used for training a SOM. Subsequently, all of the 2,003 lncRNAs or 7,148 genes were mapped to the SOM to display expression patterns. The SOMs were constructed with function *som* in the R package ‘kohonen’ (version 2.0.19, with parameters -toroidal T) [50]. A heuristic value 5 × sqrt (N) was used to set total number of map units, where N is the number of genes. The map grid was initialized with the top 10 ranked principal components of the data vectors. Training lasted for 200 iterations with the decline of learning rate vector linearly from 0.05 to 0.01 over updates. The radius was adapted toward the winning unit decreased linearly from d to 2 units, where d is a value that covers 2/3 of all unit-to-unit distances. The average relative expression of lncRNAs in each unit was assigned to hexagons for visualization with R function *polygon*. Clusters were seeded by the local minimum value of the u-matrix. Other neighbor units were then assigned to clusters according to the minimum vector distance to a seed unit. The lists of clustered genes were submitted to the Gene Set Enrichment Analysis server [23] (GSEA, http://www.broadinstitute.org/gsea/index.jsp) in order to determine enriched terms.

### Stemness-differentiation gradient

Stemness signature was defined as the set of lncRNAs whose expression was higher in GSC cultures compared with the corresponding differentiated glioma cell (DGC) cultures from three tumors (MGH26, 28 and 31). Paired *t-test* was used to call differentially expressed lncRNAs with a *p*-value threshold of 0.05 in each of the tumor-derived pairs, resulting in 31 stemness signature lncRNAs. We used the average relative expression of those lncRNAs minus the average relative expression of 2,003 lncRNAs to define a stemness score for each single cell from the tumors. Subsequently, we randomly sampled a hundred sets of lncRNAs with the same size as the stemness signature and used these random lncRNA sets to score stemness. Finally, we compared the standard deviation of stemness gradient (defined as the stemness-score profile of individual single cells within one tumor population) with the random lncRNA sets.

### Subtype analysis

The list of subtype identifier lncRNAs was collected from [16]. Thirty-one lncRNAs with the greatest variance among all single cells were used for subtype analysis. We used the average relative expression of each set of subtype predictor lncRNAs minus the average relative expression of 2,003 lncRNAs to assign an initial subtype score for each cell. To estimate the significance of the subtype scores, we randomly sampled a hundred sets of genes with the same size as each subtype predictor and used these random sets with the same procedure to define a 1% cutoff for the expected subtype scores. Finally, we classified the single cells based on the identity of the subtypes for which they passed the 1% threshold.

## SUPPLEMENTARY FIGURES AND TABLES


