# Supplementary material for: Systematic characterization of lncRNAs' cell-to-cell expression heterogeneity in glioblastoma cells
Source: Oncotarget. 2016 Feb 23;7(14):18403–14. doi: 10.18632/oncotarget.7580 (PMC4951297; doi:10.18632/oncotarget.7580)
Supplement: Supplementary file 1 [file oncotarget-07-18403-s001.pdf]

SUPPLEMENTARY FIGURES AND TABLES

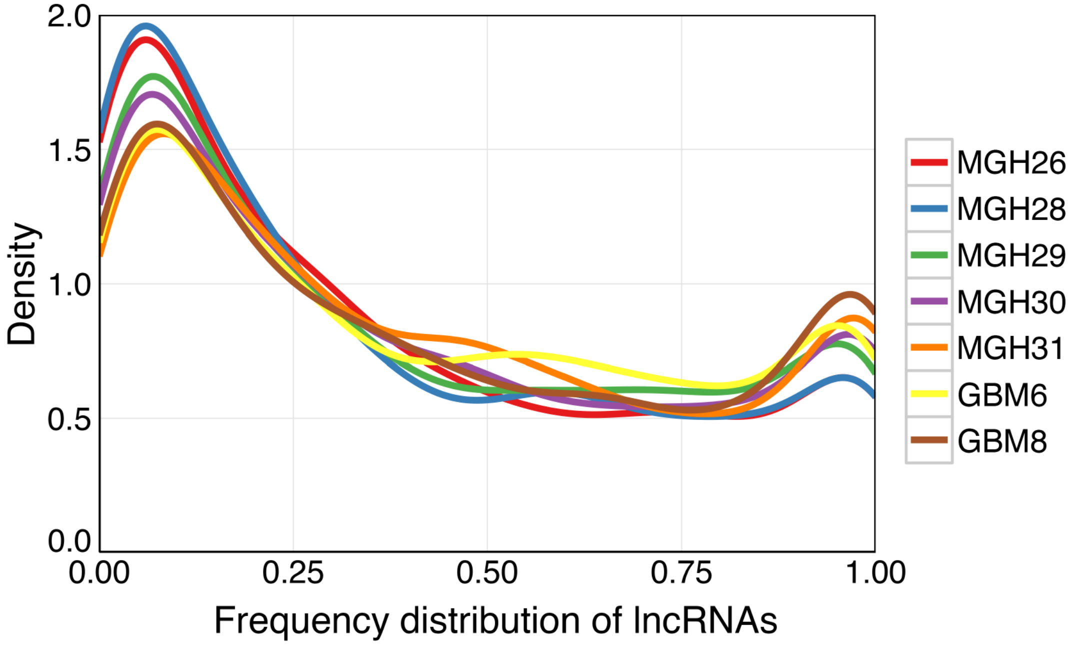

Supplementary Figure S1: Frequency distribution of individual lncRNAs in single cells of each tumor. For each tumor population, all lncRNAs expressed at least in one cell were used to calculate the expression density. The peaks at about 0.1 on the *x* axis well displayed the intratumoral heterogeneity for all five tumor samples.

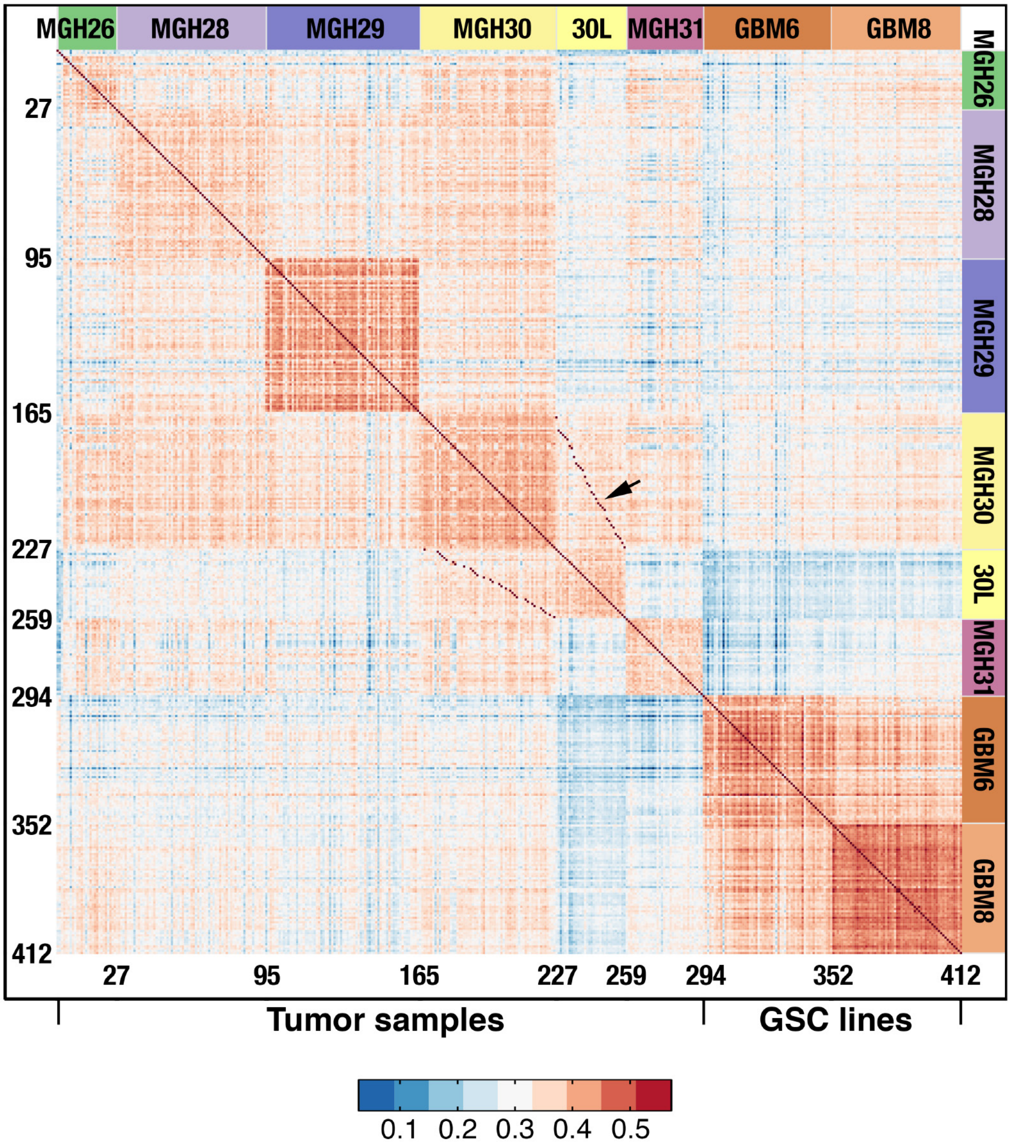

Supplementary Figure S2: Similarity and variation of lncRNA profiles in single cells. Correlation matrix for all 380 cells, ordered by five tumors (MGH26, 28, 29, 30, and 31) and two GSC lines (GBM6 and GBM8). MGH30L represents the lncRNA profile set of resequenced MGH30 libraries with longer reads. The correlation coefficients between individual cells from the same primary tumor are higher than those between cells from different tumors. GBM6 and GBM8 show higher intra- and inter-group correlation than that of tumor cells. Arrow highlights the most highly correlated lncRNA profiles, which correspond to the same MGH30 library sequenced with different read lengths.

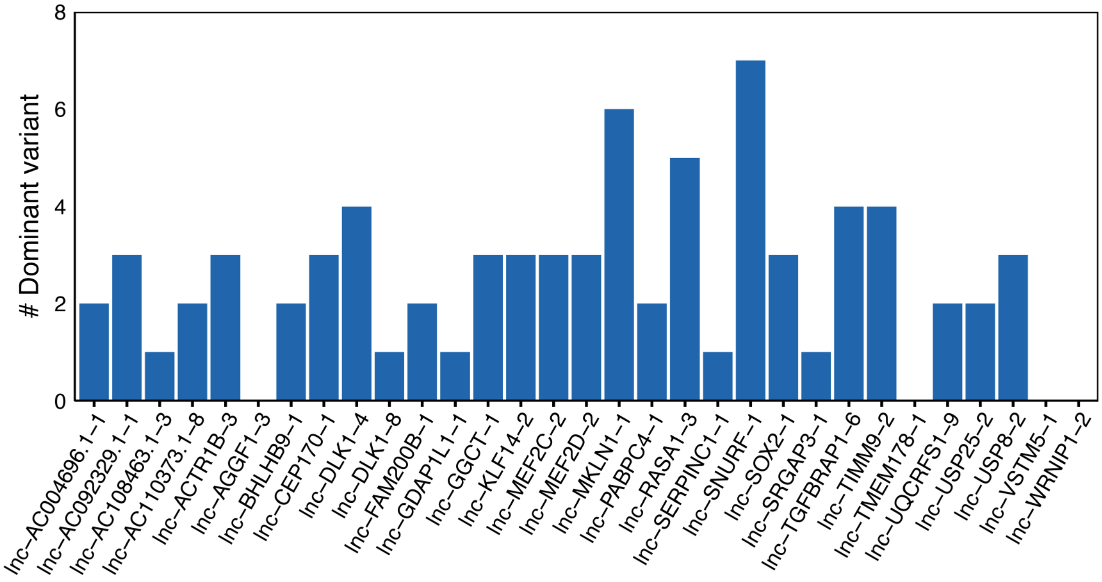

Supplementary Figure S3: Number of dominant variant for each of the 31 lncRNAs expressed in the 32 single cells from MGH30. The splice variant expressed in more than 11 single cells is defined as the dominant variant. There are 15 lncRNAs (~48% of all 31 lncRNAs) with more than two dominant variants.

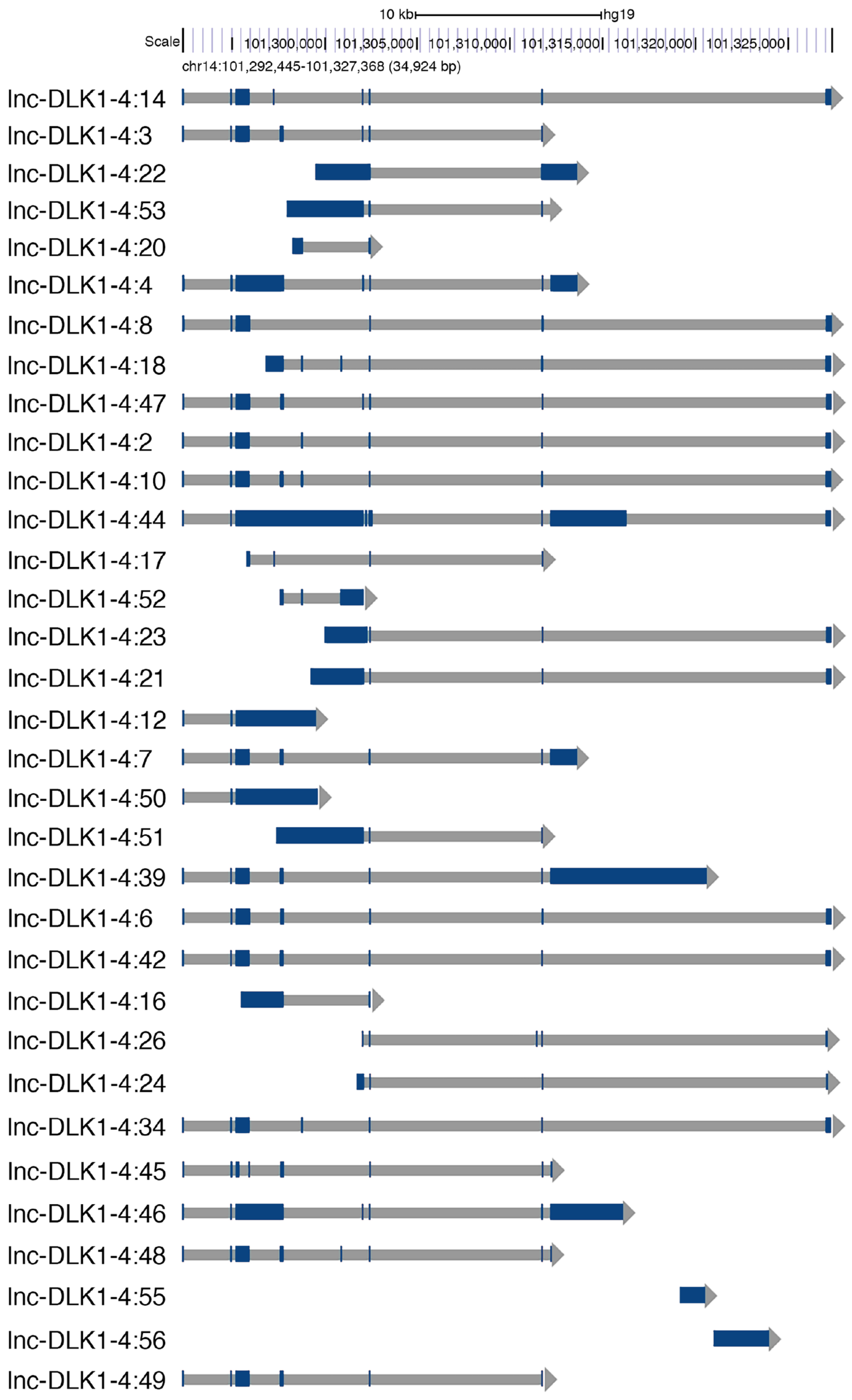

Supplementary Figure S4: Thirty-three types of *lnc-DLK1-4* splice variants detected in MGH30 tumor. Blue boxes and grey lines represent exons and introns, respectively. The schematic diagram for these variants was adapted from the LNCipedia database (http://www.lncipedia.org/db/gene/lnc-DLK1-4).

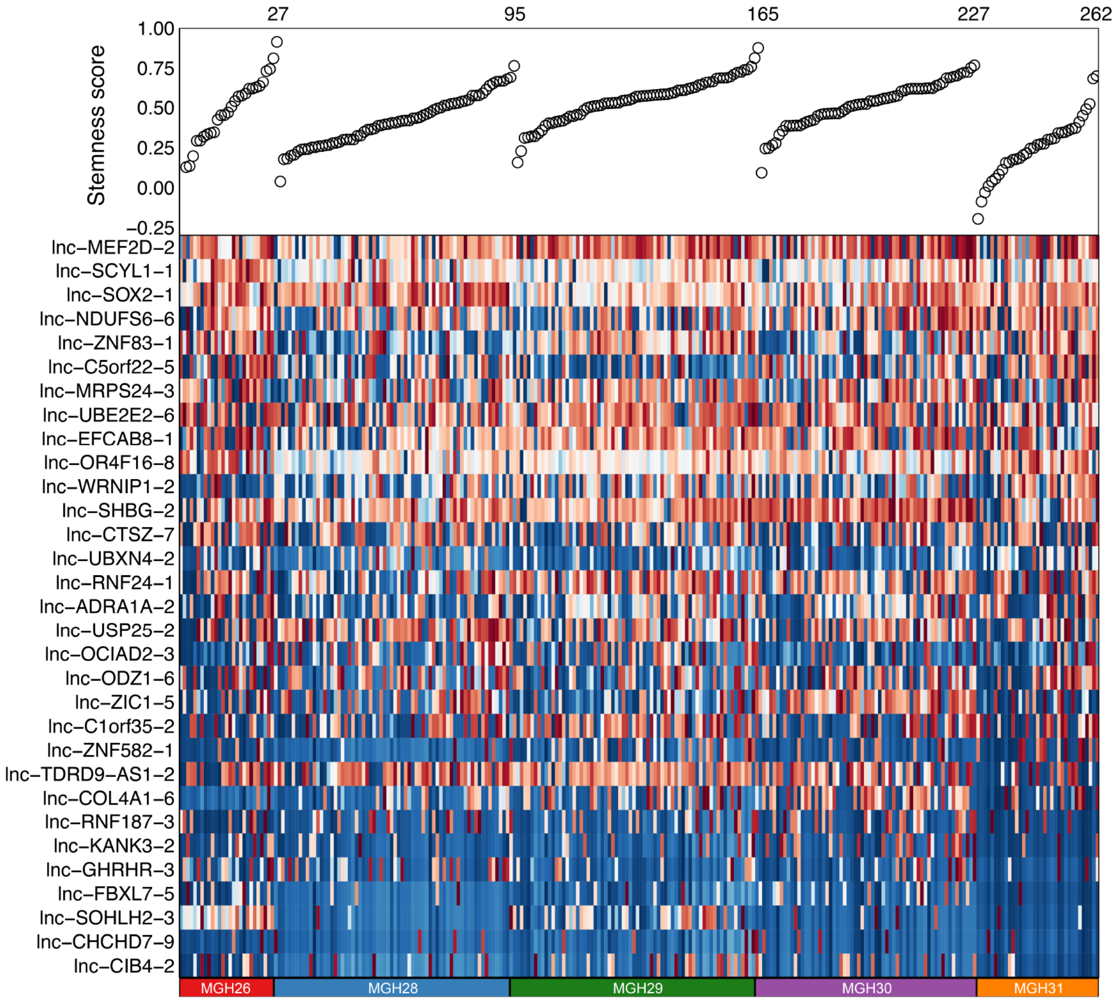

Supplementary Figure S5: Stemness signature of all single cells from five tumors. Heatmap depicts heterogeneous expression of the 31 lncRNAs standing for the stemness signature in all 262 individual cells from the five primary tumors. Plot depicts stemness scores corresponding to individual cells from each tumor.

Supplementary Table S1: Statistics on tumor samples used in our analysis

Sample number	Total cells	Selected cells	Avg % reads mapped to transcriptome	Avg # aligned reads per cell (sequencing depth ×)
MGH26	192	27	19.46	705,016 (1.17)
MGH28	96	68	21.80	791,407 (1.37)
MGH29	96	70	39.23	1,216,775 (2.07)
MGH30	96	62	26.47	1,087,709 (1.86)
MGH31	96	35	11.58	539,204 (0.83)
MGH30L	96	32	20.94	608,127 (1.01)
GBM6	96	59	33.84	1,791,420 (2.94)
GBM8	96	59	21.91	832,126 (1.37)

Supplementary Table S2: Eight clusters of coordinately expressed genes in the transcriptome SOMs

Cluster	Protein-coding genes	LncRNAs
Cluster 1	AC010441.1, ACPL2, AK4, ANKRD17, AP3B1, ARHGAP5, ARID2, ATG12, ATP13A4, BAG4, BBS4, BPTF, C12orf76, C1orf27, CCDC80, CCPG1, CHD4, CKB, CLDN12, CSNK2A2, CST3, CWC27, DDAH1, DHX29, DNAJC18, DNAJC3, EGFR, ETF1, EXTL2, FAM120B, FAM178A, FAM221A, FKBP9, FNBP1L, FUS, GCDH, GOLGB1, HNRNPM, HSD17B4, INTS12, KBTBD2, KIAA1033, KIF1B, KPNA6, KTN1, LCLAT1, LRRN3, MAGT1, MARCH6, MDM4, MEIS1, MIER1, NECAP1, NKTR, NLGN1, NUDT3, OXR1, PPP6R3, PRRC2C, PTGR1, PTPN12, RAB8A, RAD50, RBM22, RBM4B, RFC1, RFTN2, RHOBTB3, RNF5, S100PBP, SLC25A36, SLC30A7, SLC35F1, SLU7, SNX13, SPTAN1, TBC1D23, TFIP11, TMUB2, TNPO1, TOP2B, TOR1AIP2, TRIM26, TRIP11, TTC19, UPF3A, USP15, USP22, USP47, WDR33, YAF2, ZC3H13, ZNF148, ZNF43, ZNF559, ZNF644, ZNF721	lnc-AC106017.3.1-3, lnc-ARF6-3, lnc-DNMT1-2, lnc-GJD2-4, lnc-HMGA1-3, lnc-IQCF6-2, lnc-KIAA1239-1, lnc-MRPS24-3, lnc-MRPS25-1, lnc-RP11-770J1.5.1-1, lnc-TARDBP-3, lnc-TAS2R1-24, lnc-TREX1-2
Cluster 2	AL117190.2, ARL4A, ATP6V0E2, BCAT1, BSCL2, C11orf49, C16orf45, CD82, ELOVL2, GRIA2, NLGN3, NREP, RAMP1, SNRPN, SNURF, TRIB2	lnc-ANKRD20A1-7, lnc-DLK1-4(MEG3), lnc-RTL1-13, lnc-SNURF-1, lnc-TGFBRAP1-6
Cluster 3	AGT, C6orf62, DNAJB1, FOS, HEPN1, HOPX, IFITM3, ITGB8, METTL7B, MT3, NRCAM, SLC1A3, SOD2, SPARCL1, SPP1, TSPAN7, UBB	lnc-ITGB8-5, lnc-SCYL1-1(MALAT1), lnc-SHBG-2, lnc-TIMM9-2, lnc-WNT16-4, lnc-ZDHHC21-5
Cluster 4	ACTR10, ARCN1, ARFGAP2, ARL1, ASNA1, ATP6V1D, ATRX, BECN1, BEX4, CCDC53, CERS5, CPNE3, CTSL, DPF2, FAM162A, LSM10, MLLT11, MPC1, MTFR1L, MTRNR2L1, MYL6B, NCAM1, NFIA, NOVA1, NRSN2, PCDH9, PDE4B, PEA15, PLOD1, PPA2, PPP2R2B, PRR13, RAC1, RASSF2, RNF14, RSRC2, S100A13, TCF4, TM2D1, TMEM219, TMEM50A, TMEM50B, TRAPPC1, TRAPPC3, TRO, TSG101, TXLNA, TXNDC15, UBE2A, USP4, VPS41, WLS, YWHAB	lnc-FAM177B-1, lnc-FAM72B-11, lnc-OPA1-6, lnc-TXNDC15-1, lnc-WBSCR16-3

(*Continued*)

Cluster	Protein-coding genes	LncRNAs
Cluster 5	ACTR6, ADAM10, ADAR, AKIP1, ALAS1, ATP6V1A, ATXN7L3B, BCL2L13, C12orf65, C3orf14, CD58, CERS2, CHORDC1, CNOT1, CNPPD1, CREB3, DHRS7B, DHX8, DNAJC2, DPM2, DTWD1, DUSP11, DYNC1LI1, DYNLT3, EAPP, ERP44, FAM103A1, FAM115A, FH, GDAP1, GFM1, GPATCH2L, HIAT1, IFI27L1, IGBP1, IQGAP1, MED8, MESDC2, MOB1A, MRPL48, MRPS12, MRPS6, MTX1, NBN, NDUFAF1, NDUFV3, NRAS, NUDT9, OSER1, PAFAH1B1, PEG10, PEX11B, PIGF, PMS1, PMS2, POGLUT1, POLR2C, PPP2R3C, PPP6C, PRRC1, RBM48, RHEB, RHOJ, RPF1, SCAMP2, SERPINE1, SLC31A1, SLC39A10, SLC39A9, SPATS2L, STAU2, TAF12, TCEB3, TIMM22, TIPRL, TMEM126B, TMEM38B, TMEM68, TMX3, TNPO3, TOR1AIP1, TYW1, UFSP2, UTP6, VPS45, WDR3, ZCCHC10, ZFAND2A, ZNF880	lnc-AP1M2-1, lnc-CTDSP2-2, lnc-TPTE-1, lnc-UPK3B-1
Cluster 6	APOD, CA14, CD163L1, CHIC2, CHRDL1, CPM, CSAG1, CYTL1, EIF4A3, EPHA3, ERBB3, FABP3, FKBP7, GAD1, IL13RA2, INPP4B, MIA, PIR, SH3KBP1, SLC29A1, SLC38A1, SLCO4A1, ST3GAL6, TM4SF1, TMEM98, TMSB15A	lnc-AC233263.1-7, lnc-AP000525.1-3, lnc-C5orf17-8, lnc-CD163-3, lnc-CHIC2-2, lnc-CHIC2-3, lnc-CTNNA2-4, lnc-DDX1-3, lnc-NAP1L2-1, lnc-NBAS-4, lnc-NRG1-2, lnc-POTEG-4, lnc-POTEM-2, lnc-VSIG7-1
Cluster 7	ATP2B4, ANXA4, ASL, BAALC, C21orf119, CAPN2, DNPEP, HAS2, IMMP2L, MYC, SPRYD3, SULF2, THBS3, UPP1	lnc-LRR1-1, lnc-PTS-1, lnc-STYXL1-2, lnc-ZNF644-1
Cluster 8	ADM, ARSJ, ATF3, C10orf10, C15orf52, C19orf66, CAMK2N1, CCDC102B, CD68, CHRNA9, CITED1, CLEC2B, CP, CTGF, CTSK, CXADR, CXCL14, DDIT4L, DDRGK1, DDX3Y, DPY19L1, DUSP1, EIF1AY, ELMOD1, GFPT2, GPRC5A, GPX3, IGFBP5, IRF1, KRBOX1, MID1, MVP, MYADM, MYL9, MYOF, NCOA7, P4HA2, PAM, PCSK1, PGM2L1, PNRC1, PRICKLE1, PROX1, RAB30, RASSF8, RDH10, ROM1, SDC4, SELM, SERTAD1, SIAE, SLC40A1, SLN, SPOCD1, SRPX, SRPX2, SSX2IP, TMEM144, TMEM176B, TREM1, VEGFA, ZYX	lnc-ARF6-1, lnc-ARRDC3-1, lnc-ATAD2-1, lnc-ATL3-2, lnc-ATP6V0A1-1, lnc-COL4A1-2, lnc-DPP6-6, lnc-FAM198A-3, lnc-FBXO45-4, lnc-FDFT1-1, lnc-MFSD9-14, lnc-SIPA1L1-2, lnc-SRPX2-1, lnc-TLCD2-1, lnc-USP9Y-1

Supplementary Table S3: Thirty-one differentially expressed lncRNAs defined as the stemness signature

Name in LNCipedia 3.0	Alias	*p*-value
lnc-SCYL1-1	MALAT1; ENSG00000251562; OTTHUMG00000166322.1	5.33E-03
lnc-C1orf35-2	N/A	5.67E-03
lnc-NDUFS6-6	RP11-259O2.3; XLOC_004251; linc-C5orf38-6; ENSG00000249731; OTTHUMG00000161603.1	8.81E-03
lnc-UBE2E2-6	N/A	1.01E-02
lnc-SHBG-2	N/A	1.08E-02
lnc-COL4A1-6	N/A	1.25E-02
lnc-SOHLH2-3	N/A	1.43E-02
lnc-TDRD9-AS1-2	N/A	1.62E-02
lnc-CTSZ-7	N/A	1.95E-02
lnc-UBXN4-2	AC093391.2; ENSG00000231890; OTTHUMG00000153578.1	2.42E-02
lnc-MEF2D-2	N/A	2.49E-02
lnc-OR4F16-8	N/A	2.66E-02
lnc-WRNIP1-2	GMDS-AS1; ENSG00000250903; RP1-80B9.2; OTTHUMG00000014120.3	2.76E-02
lnc-EFCAB8-1	RP5-1085F17.3; ENSG00000260257; OTTHUMG00000177126.1	2.77E-02
lnc-ZNF582-1	linc-ZNF582; XLOC_013415	2.97E-02
lnc-C5orf22-5	N/A	3.23E-02
lnc-CIB4-2	N/A	3.27E-02
lnc-ADRA1A-2	N/A	3.44E-02
lnc-USP25-2	LINC00478; ENSG00000215386; C21orf34; OTTHUMG00000074377.1	3.54E-02
lnc-ODZ1-6	N/A	3.85E-02
lnc-GHRHR-3	N/A	3.88E-02
lnc-CHCHD7-9	N/A	3.91E-02
lnc-RNF187-3	N/A	4.02E-02
lnc-ZNF83-1	AC010332.1; ENSG00000246015	4.28E-02
lnc-KANK3-2	RAB11B-AS1; ENSG00000269386	4.34E-02
lnc-OCIAD2-3	N/A	4.39E-02
lnc-RNF24-1	ENSG00000247957	4.40E-02
lnc-MRPS24-3	N/A	4.71E-02
lnc-FBXL7-5	N/A	4.76E-02
lnc-SOX2-1	SOX2-OT; ENSG00000242808; RP11-4B14.2; OTTHUMG00000158186.2	4.79E-02
lnc-ZIC1-5	linc-AGTR1-4 ;XLOC_002859 ;	4.88E-02

Supplementary Table S4: Four classifier lncRNA sets for GBM subtypes

Subtypes	Name in LNCipedia 3.0	Alias
Proneural (PN)	lnc-BHLHB9-1	ENSG00000223546
	lnc-C2orf42-3	ENSG00000179818
	lnc-MEF2C-2	ENSG00000245526
	lnc-SCRG1-1	XLOC_004165
	lnc-SNURF-1	ENSG00000257151
	lnc-SRGAP3-1	ENSG00000206573
	lnc-SRGAP3-1	ENSG00000254485
	lnc-VAMP1-1	ENSG00000215039
	lnc-ZSCAN10-3	ENSG00000263072
Neural (N)	lnc-ABCB4-1	ENSG00000182165
	lnc-GDAP1L1-1	ENSG00000223891
	lnc-IL23A-2	ENSG00000257303
	lnc-KLF14-2	ENSG00000231721
	lnc-RASA1-3	ENSG00000247828
	lnc-RNF151-1	ENSG00000255198
	lnc-SLC30A7-2	ENSG00000233184
	lnc-SPG11-1	ENSG00000179523
	lnc-TMEM116-2	ENSG00000234608
	lnc-TOP1MT-2	ENSG00000253716
	lnc-USP6-1	ENSG00000234327
Mesenchymal (MES)	lnc-DISP1-1	ENSG00000228106
	lnc-EIF6-1	ENSG00000126005
	lnc-NRG1-2	ENSG00000247134
Classical (CL)	lnc-AC006035.2-2	ENSG00000229108
	lnc-C11orf55-1	ENSG00000254919
	lnc-COL4A1-2	XLOC_010719
	lnc-NTSR2-5	ENSG00000225649
	lnc-RALGAPB-1	ENSG00000174365
	lnc-RBM48-1	ENSG00000237819
	lnc-TGFBRAP1-6	ENSG00000233639
	lnc-ZIC1-5	ENSG00000239922
